# Long-Term Outcomes of Children and Adolescents With Postural Tachycardia Syndrome After Conventional Treatment

**DOI:** 10.3389/fped.2019.00261

**Published:** 2019-06-27

**Authors:** Chunyan Tao, Wenxin Lu, Jing Lin, Hongxia Li, Xueying Li, Chaoshu Tang, Junbao Du, Hongfang Jin

**Affiliations:** ^1^Department of Pediatrics, Peking University First Hospital, Beijing, China; ^2^Department of Medical Statistics, Peking University First Hospital, Beijing, China; ^3^Department of Physiology and Pathophysiology, Peking University Health Science Center, Beijing, China; ^4^Key Laboratory of Molecular Cardiovascular Science, The Ministry of Education, Beijing, China

**Keywords:** postural tachycardia syndrome, conventional interventions, long-term outcomes, children, adolescents

## Abstract

**Objectives:** To explore the long-term outcomes of children and adolescents with postural tachycardia syndrome receiving conventional interventions.

**Materials and Methods:** A total of 121 patients were recruited, but 6 (5.0%) of them were lost at follow-up. The detailed clinical data were collected, and the reoccurrence and frequency of orthostatic intolerance symptoms were evaluated with a mean followed-up period of 18.7 months (range, 14–74 months). The Kaplan-Meier curve was used to show the cumulative symptom-free rate of patients over time. Factors influencing the long-term outcomes were examined using the Cox's proportional hazards models.

**Results:** The cumulative symptom-free rate was gradually increased over time. It was 48.4% at the 1-year follow-up and increased to 85.6% at the 6-year follow-up. The duration of symptoms before treatment and the maximum upright heart rate in standing-up test were identified as independent indicators for the long-term outcomes. Each 1-month prolongation in the duration of symptoms before treatment was associated with a 1.2% decrease in the cumulative symptom-free rate. However, each 1-bpm increase in the maximum upright heart rate in standing-up test was associated with a 2.1% increase in the cumulative symptom-free rate.

**Conclusions:** The long-term outcomes of postural tachycardia syndrome patients who received conventional interventions are benign and the cumulative symptom-free rate was gradually increased over time. The prolonged duration of symptoms before treatment and the reduced maximum upright heart rate in standing-up test are the independent risk indicators.

## Introduction

Postural tachycardia syndrome (POTS) is an important form of orthostatic intolerance (OI) and is a constellation of symptoms including lightheadedness, chest discomfort, blurred vision, palpitations, and even syncope, which are elicited by excessive upright tachycardia and relieved by recumbence (1).

POTS in adolescents was firstly described by Bou-Holaigah et al. and it occurs at an estimated rate of 6.8% (2, 3). It negatively affects their physical and psychological health and impairs their quality of life (4, 5). Therefore, the investigation of the treatment for POTS has attracted great interest of clinical scientists. Up to now, multiple therapeutic methods have been introduced to deal with POTS in children and adolescents (6), among which conventional interventions are irreplaceable for their simplicity and for no obvious adverse effects (7, 8). There have been several studies on the therapeutic efficacy of conventional interventions in pediatric POTS patients (9–14). However, their long-term outcomes have never been reported, which limits pediatricians to predict the prognosis of this disease.

Therefore, this study focuses on the long-term outcomes and the associated factors of pediatric POTS patients receiving conventional treatment.

## Materials and Methods

We enrolled 121 patients [54 males and 67 females, median age 12.0 (10.0, 13.0) years] with a diagnosis of POTS and treated with conventional interventions at the Department of Pediatrics of Peking University First Hospital from July 2012 to July 2017. One hundred and fifteen patients completed our follow-up by telephone or clinic visits, but the remaining six patients (5.0%) were lost. This research was authorized by the Ethics Committee of Peking University First Hospital and all guardians of included patients gave written informed consent in accordance with the percepts expressed in the Declaration of Helsinki.

Mean corpuscular hemoglobin concentration and platelet were from blood routine test. Blood was drawn by venipuncture after at least 4 h fast and collected in a tube containing ethylene diamine tetraacetic acid. It was tested immediately at the Medical Laboratory of Peking University First Hospital (XE-5000, SYSMEX Corporation, Kobe, Japan) (13).

A urine collection kit for each patient was prepared in advance. A 24 h urine sample was collected and females were asked to take the 24 h urine samples during non-menstrual days. The 24 h urinary sodium excretion was determined using the ion-selective electrode method (Cobas 6000, Roche, Switerland). Twenty-four hours urinary sodium excretion = the concentration of sodium × the total urine volume (15).

Drugs influencing autonomic nervous function were avoided for at least 3 days before standing-up test. The testing environment was quiet, warm and dimly lit. Heart rate and blood pressure were recorded after 10–20 min of supine rest. Then, the patient was asked to stand for another 10 min, with simultaneously monitoring of heart rate and blood pressure (Dash 2000, General Electric, Schenectady, New York). The test was terminated ahead if the patient could not persist in finishing it. ΔHR = the maximum heart rate during standing—the baseline heart rate during supine (16).

Criteria of POTS in children and adolescents consisted of the following: (1) the presence of predisposing factors such as prolonged standing, rapid postural changes or exposure to emotional stress; (2) suffering from OI symptoms; (3) a positive response during standing-up test [normal heart rate in the supine position and an increment of heart rate ≥ 40 bpm or a maximum heart rate ≥ 130 bpm (in children aged 6–12 years) or ≥ 125 bpm (in adolescents aged 13–18 years) in standing-up test, without a decrease in blood pressure ≥20/10 mmHg]; and (4) the exclusion of other diseases that may likely cause OI symptoms (16).

The symptom scores of POTS were determined by the presence of typical symptoms (dizziness, headache, syncope, blurred vision, palpitation, chest discomfort, nausea, tremors, and sweating). Each symptom was counted based on its frequency (0 score, no symptom; 1 score, each symptom occurring once per month; 2 scores, 2–4 times per month; 3 scores, 2–7 times per week; 4 scores, more than once per day) and the total scores were calculated by summing all of the scores of each symptom (17).

Conventional interventions were used for each patient once diagnosed with POTS. Details of conventional interventions were listed as follows: (1) health education: informing patients and their guardians of possible causes, and teaching them how to avoid triggers and to protect themselves when experiencing OI symptoms; (2) supplement of water and salt: one bag of oral rehydration saline (Anjian Pharma Company, Xi‘an, China) per day, containing 0.650 g sodium chloride, 0.725 g sodium citrate, 0.375 g potassium chloride and 3.375 g anhydrous glucose; (3) orthostatic training: asking patients to stand against a wall with the feet 15 cm away from the wall for a gradually increasing duration from 3 to 30 min, 2–3 times a day, depending on their orthostatic tolerance (11, 18). None of the patients continued implementing these interventions during the long-term follow-up. However, two of them got metoprolol or midodrine hydrochloride treatment for recurrent OI symptoms and they were regarded as censored subjects at the beginning of the introduction of the abovementioned new interventions. Designated doctors conducted the follow-up tasks by telephone or clinic visits in October 2018, with a mean follow-up duration of 18.7 months (range, 14–74 months). The main content of follow-up contained the reoccurrence and frequency of OI symptoms.

SPSS version 21.0 (IBM, Armonk, New York) was used for all data analyses. The normality of continuous data was examined by the Shapiro-Wilk test and continuous data with normal distribution are presented as mean ± standard deviation, otherwise as median (P_25_, P_75_). Bivariate correlations for continuous variables were tested using Pearson's correlation coefficient. Categorical variables are summarized as numbers (percentages). No reoccurrence of OI symptoms during follow-up was defined “event.” The Kaplan-Meier curve was used to show the trend of cumulative symptom-free rate of patients over time. The prognostic significance of baseline demographics, clinical characteristics, personal history, family history about OI, characteristics during standing-up test, blood routine test parameters and 24 h urinary sodium excretion with respect to long-term outcomes was examined using the Cox's proportional hazards models. Parameters with a *p*-value <0.1 in univariate model were introduced in the multivariate model by a stepwise method. All *p*-values were 2-sided and a *p*-value <0.05 was considered statistically significant.

## Results

A total of 121 pediatric POTS patients receiving conventional treatment were enrolled in this research, but 6 of them were lost at follow-up (5.0%). Among the 115 followed-up cases [50 males and 65 females, median age 12.0 (10.0, 13.0) years], 77 cases (67.0%) were accompanied with predisposing factors (prolonged standing as the most common one, then postural changes) and 61 cases (53.0%) suffered from syncopal events. A varied period of treatment, 3.5 (3, 5) months, was mainly because of the difference in the time of re-visiting our clinics and the more details are presented in Table 1.

**Table 1:** Baseline demographic and clinical characteristics of the study participants.

**Characteristics**	**Values**
Participants (*n*)	115
Age (years)	12.0 (10.0, 13.0)
Gender (n, male/female)	50/65
Body mass index (kg/m^2^)	17.4 (16.1, 19.8)
Duration of symptoms before treatment (months)	10.5 (2.0, 36.0)
**MEDICAL HISTORY**	
Predisposing factors (n, yes/no)	77/38
Syncope (n, yes/no)	61/54
Symptom scores before treatment (points)	4 (3, 7)
**PERSONAL HISTORY**	
Motion sickness (n, yes/no)	16/99
Allergic diseases (n, yes/no)	29/86
Family history about OI (n, yes/no)	29/86
**CHARACTERISTICS IN STANDING-UP TEST**	
Supine heart rate (bpm)	74 ± 11
Maximum upright heart rate (bpm)	121 ± 14
Changes of heart rate (bpm)	46 (40, 53)
**ITEMS OF BLOOD ROUTINE TEST**	
Mean corpuscular hemoglobin concentration (g/L)	347 (339, 354)
Platelet (^*^10^9^/L)	257 (229, 298)
24 h urinary sodium excretion (mmol/24 h)	102 (73, 135)
Duration of treatment (months)	3.5 (3, 5)

*OI, orthostatic intolerance. Continuous data with normal distribution are presented as mean ± standard deviation, otherwise as median (P_25_, P_75_) and categorical variables are summarized as numbers*.

**Figure 1:**
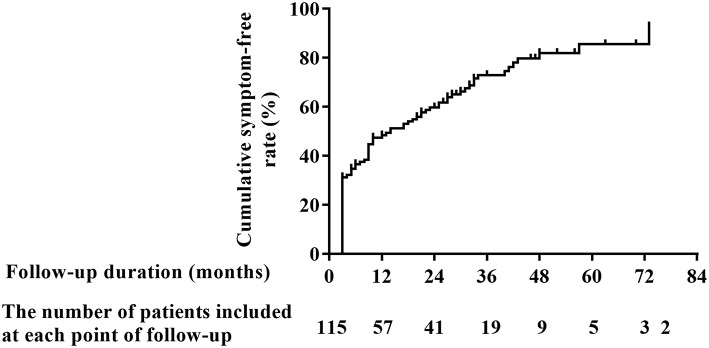
Cumulative symptom-free rate of POTS patients receiving conventional interventions during long-term follow-up. POTS, postural tachycardia syndrome.

In Figure 1, the number of patients with OI symptoms was gradually decreased over time, with a range of duration from 14 to 74 months to demonstrate the patients' outcomes at varied follow-up time. The cumulative symptom-free rates at 1, 2, 3, 4, 5, and 6 years after the beginning of follow-up were 48.4, 59.8, 73.0, 82.0, 85.6 and 85.6%, respectively.

Parameters with a *p* < 0.1 in the Cox univariate analysis (the duration of symptoms before treatment, the supine heart rate and the maximum heart rate in standing-up test, Table 2) were introduced in the multivariate analysis. However, given the strong collinearity between the supine heart rate and the maximum heart rate in standing-up test (*r* = 0.711, 95% CI, 0.620–0.795, *p* < 0.001) and the maximum heart rate used as one of the diagnostic criteria for POTS in standing-up test (16), only the maximum upright heart rate and the duration of symptoms before treatment were included eventually. The multivariate model showed that both of them were independent indicators for the long-term outcomes of the study patients (*p* < 0.05, Table 3). For each 1-month prolongation in the duration of symptoms before treatment, a decrease of 1.2% in the cumulative symptom-free rate was detected. However, for each 1-bpm acceleration in the maximum upright heart rate during standing-up test, an increase of 2.1% was detected.

**Table 2:** Results of univariate Cox's proportional hazard model regression.

**Characteristics**	**B**	**SE**	**Wald**	***p*-value**	**HR (95% CI)**
Age (years)	−0.067	0.045	2.164	0.141	0.936 (0.856–1.022)
Gender (male vs. female)	−0.181	0.221	0.674	0.412	0.834 (0.541–1.286)
Body mass index (kg/m^2^)	−0.033	0.043	0.582	0.445	0.968 (0.890–1.052)
Duration of symptoms before treatment (months)	−0.009	0.005	3.481	0.062	0.991 (0.982–1.000)
**MEDICAL HISTORY**					
Predisposing factors (yes vs. no)	−0.160	0.234	0.465	0.495	0.852 (0.539–1.349)
Syncope (yes vs. no)	−0.028	0.22	0.016	0.900	0.973 (0.632–1.498)
Symptom scores before treatment (points)	−0.001	0.028	0.001	0.978	0.999 (0.946–1.055)
**PERSONAL HISTORY**					
Motion sickness (yes vs. no)	0.387	0.317	1.488	0.223	1.472 (0.791–2.739)
Allergic diseases (yes vs. no)	−0.198	0.25	0.625	0.429	0.821 (0.503–1.340)
Family history about OI (yes vs. no)	0.11	0.253	0.191	0.662	1.117 (0.681–1.832)
**CHARACTERISTICS IN STANDING–UP TEST**					
Supine heart rate (bpm)	0.025	0.011	5.703	0.017	1.026 (1.005–1.047)
Maximum upright heart rate (bpm)	0.014	0.008	3.153	0.076	1.014 (0.999–1.030)
Changes of heart rate (bpm)	−0.001	0.011	0.004	0.952	0.999 (0.977–1.022)
**ITEMS OF BLOOD ROUTINE TEST**					
Mean corpuscular hemoglobin concentration (g/L)	0.011	0.009	1.437	0.231	1.011 (0.993–1.029)
Platelet (*10^9^/L)	0.002	0.002	0.757	0.384	1.002 (0.998–1.006)
24 h urinary sodium excretion (mmol/24 h)	−0.001	0.002	0.218	0.641	0.999 (0.995–1.003)
Duration of treatment (months)	−0.001	0.106	<0.001	0.989	0.999 (0.812–1.228)

*OI, orthostatic intolerance*.

**Table 3:** Results of multivariate Cox's proportional hazard model regression.

**Characteristics**	**B**	**SE**	**Wald**	***p*-value**	**HR (95% CI)**
Duration of symptoms before treatment (months)	−0.012	0.005	5.652	0.017	0.988 (0.979–0.998)
Maximum upright heart rate in standing-up test (bpm)	0.021	0.009	5.661	0.017	1.021 (1.004–1.039)

## Discussion

In this research, we found that the cumulative symptom-free rate was gradually increasing during follow-up period. The duration of symptoms before treatment and the maximum upright heart rate in standing-up test were independent indicators for the long-term outcomes of pediatric POTS patients after conventional treatment, the former being a risk factor with its prolongation and the latter being a protective factor with its acceleration.

POTS is a hot topic in pediatrics, not only for its relatively high prevalence but also for its impact on the quality of life (19). We found that although most patients had benign outcomes, some of them still sustained OI symptoms for such a long term that it would be of help to reduce their psychological burden if clinicians could predict the prognosis and its influencing factors in advance. Kimpinski et al. conducted a prospective study to demonstrate favorite clinical outcomes at the 1-year follow-up, however, their therapeutic regimens were miscellaneous (20).

Prolonged duration of symptoms before treatment was identified as a risk factor for the prognosis of POTS patients receiving conventional interventions. Namely, the earlier such treatment was carried out, the better the prognosis would be. Unfortunately, in a large pediatric sample-sized study, Boris et al. observed that most of the participants visited doctors after suffering from OI symptoms for about 2 years (21), implying that the patients and their guardians might overlook such symptoms and more importantly, the physicians might lack the awareness of POTS and make misdiagnosis sometimes (22). Our results provided physicians with the evidence to encourage patients to visit doctors as early as possible and emphasized the importance of recognizing POTS.

When changing from supine to upright, gravity produces a rapid and large downward shift from thorax into vessels of the lower body. Simultaneously, the endogenous integrated mechanisms are activated to compensate the reduced venous return. Any abnormality in the regulated progress might result in postural tachycardia (23). Our team previously found that the maximum upright heart rate in standing-up test ≥ 123 bpm could predict a favorable outcome after oral rehydration saline therapy in pediatric POTS patients (10). Tachycardia would be more obvious when reduced blood volume and impaired muscle sympathetic nerve activity occur concurrently in POTS patients, both of which are identified as common mechanisms for POTS (24, 25). Supplement of water and salt and orthostatic training could not only increase blood volume but also improve muscle sympathetic nerve activity (10, 26, 27). Under such condition, conventional treatment might improve patients' well-being significantly. It was observed in this study that the cumulative symptom-free rate would increase by 2.1% if there was a 1-bpm acceleration in the maximum upright heart rate in standing-up test. However, body mass index, mean corpuscular hemoglobin concentration, 24 h urinary sodium excretion and platelet, reported to have correlations with blood volume or autonomic nervous function (9, 10, 12, 28), were not independent indicators for the long-term outcomes for their un-representativeness of both blood volume and autonomic nervous function. Additionally, allergic diseases, common comorbidities in pediatric POTS patients (29, 30), and inheritance were not testified as independent factors.

Exactly, some limitations existed in the present study: (1) there is no control group in the present study. We could not tell the differences among patients receiving conventional interventions, pharmacological therapies and nothing; (2) there is a possibility of bias for the retrospective study design; (3) the study have the limitation of generalizability to other study populations for the characteristics, such as the similar gender ratio of the subjects in our study; and (4) the hazard ratios of the duration of symptoms before treatment and the maximum upright heart rate in the standing-up test were close to one.

To interpret the results of this study cautiously, the data showed that the cumulative symptom-free rate of POTS patients receiving conventional treatment would increase gradually over time and implementing conventional interventions to POTS patients with obvious tachycardia as early as possible might be of great help in improving their long-term outcomes.

## Data Availability

The raw data supporting the conclusions of this manuscript will be made available by the authors, without undue reservation, to any qualified researcher.

## Ethics Statement

This research was authorized by the Ethics Committee of Peking University First Hospital and all guardians of included patients gave written informed consent in accordance with the percepts expressed in the Declaration of Helsinki.

## Author Contributions

CyT had primary responsibility for the protocol development, patient enrollment, data collecting, preliminary data analysis, and writing the manuscript. WL, JL, and HL assisted with data collecting and preliminary data analysis. XL gave important advice on study design and revised data analysis. CsT gave important advice on the subject and revised the manuscript. JD and HJ supervised the design and execution of the study, checked the data analysis, contributed to the writing of the manuscript, and had a final approval of the manuscript submitted. All authors have read and approved the final manuscript and assumed full responsibility for its contents.

### Conflict of Interest Statement

The authors declare that the research was conducted in the absence of any commercial or financial relationships that could be construed as a potential conflict of interest.

## Abbreviations

POTS: Postural tachycardia syndrome
OI: Orthostatic intolerance.
